# Quantifying the Integration of Quorum-Sensing Signals with Single-Cell Resolution

**DOI:** 10.1371/journal.pbio.1000068

**Published:** 2009-03-24

**Authors:** Tao Long, Kimberly C Tu, Yufang Wang, Pankaj Mehta, N. P Ong, Bonnie L Bassler, Ned S Wingreen

**Affiliations:** 1 Department of Physics, Princeton University, Princeton, New Jersey, United States of America; 2 Department of Molecular Biology, Princeton University, Princeton, New Jersey, United States of America; 3 Howard Hughes Medical Institute, Chevy Chase, Maryland, United States of America; Harvard University, United States of America

## Abstract

Cell-to-cell communication in bacteria is a process known as quorum sensing that relies on the production, detection, and response to the extracellular accumulation of signaling molecules called autoinducers. Often, bacteria use multiple autoinducers to obtain information about the vicinal cell density. However, how cells integrate and interpret the information contained within multiple autoinducers remains a mystery. Using single-cell fluorescence microscopy, we quantified the signaling responses to and analyzed the integration of multiple autoinducers by the model quorum-sensing bacterium Vibrio harveyi. Our results revealed that signals from two distinct autoinducers, AI-1 and AI-2, are combined strictly additively in a shared phosphorelay pathway, with each autoinducer contributing nearly equally to the total response. We found a coherent response across the population with little cell-to-cell variation, indicating that the entire population of cells can reliably distinguish several distinct conditions of external autoinducer concentration. We speculate that the use of multiple autoinducers allows a growing population of cells to synchronize gene expression during a series of distinct developmental stages.

## Author Summary

Although bacteria are unicellular, the individual cells communicate with each other via small diffusible molecules. This communication process, known as quorum sensing, allows groups of bacteria to track the density of the population they are in, synchronize gene expression across the population, and thereby carry out collective activities similar to those of cells in multi-cellular organisms. Many bacterial species use multiple signaling molecules, but it remains a mystery why multiple signals are required and how the information encoded in them is integrated by bacteria. To explore these questions, we studied a model quorum-sensing bacterium Vibrio harveyi. Using single-cell fluorescence microscopy, we quantified quorum-sensing responses and analyzed the mechanism of integration of multiple signals. Surprisingly, we found that information from two distinct signals is combined strictly additively, with precisely equal weight from each signal. Our results revealed a coherent response across the population with little cell-to-cell variation, allowing the entire population of bacterial cells to reliably distinguish multiple environmental states. We argue that multiple signals and multiple response states could be used to distinguish distinct stages in the development of a bacterial community.

## Abstract 3

How do bacteria integrate multiple cell-to-cell communication signals? Information from two signaling molecules is combined in a strictly additive way with equal weight from each signal.

## Introduction

In a process called quorum sensing, bacteria communicate with one another using extracellular signaling molecules called autoinducers. Quorum sensing allows groups of bacteria to track their cell numbers, synchronize gene expression on a population-wide scale, and thereby carry out collective activities. In quorum sensing, bacteria produce, release, and detect autoinducers that accumulate in a cell-density–dependent manner, and, thus, autoinducer concentration serves as a proxy for cell number. Quorum-sensing systems are widespread in the bacterial world, existing in both Gram-negative and Gram-positive bacteria, and quorum sensing is used to control such diverse functions as bioluminescence, virulence-factor secretion, biofilm formation, conjugation, and antibiotic production [1–3].

Typically, Gram-negative bacteria use acyl-homoserine lactones and Gram-positive bacteria use peptides as autoinducers. To our knowledge, these two kinds of molecules most often promote intraspecies cell–cell communication, because a particular acyl-homoserine lactone or particular peptide can be detected only by the bacterial species that produces it [2]. In addition, a non–species-specific autoinducer called AI-2, which is a family of interconverting molecules all derived from the same precursor 4,5-dihydroxy 2,3-pentanedione, is produced and detected by a large variety of both Gram-negative and Gram-positive bacteria [4,5]. Interestingly, many bacterial species use more than a single autoinducer molecule for quorum sensing. For example, Gram-negative bacteria (e.g., *Rhizobium*) can use multiple homoserine lactones and likewise, Gram-positive bacteria (e.g., *Bacillus*) can use several peptides for communication [2,6]. These bacteria have evolved sophisticated quorum-sensing circuits to detect and integrate the information contained in multiple autoinducers.

It remains a mystery how and why bacteria integrate multiple autoinducer signals and what additional information multiple autoinducers reveal about the cells' environment that one autoinducer cannot reveal [7]. Furthermore, while in principle, quorum sensing enables bacteria to act in synchrony, the behavior of the entire population is ultimately dictated by events inside single cells. Recent single-cell studies of gene expression in bacteria have revealed that noise is inevitable even for isogenic cells in essentially homogeneous environments, and that noise can result in heterogeneous phenotypes within a population [8–14]. Likewise, in quorum sensing, noise could make individual cells behave differently from one another even if they receive identical autoinducer inputs. To understand quorum-sensing signal integration and, ultimately, the evolution of cooperative behaviors at the population level, it is imperative to understand how cells behave individually. Specifically, do cells respond in unison or do they maintain population diversity? Bulk measurements—which focus on the population's response—generally mask the behavior of individual cells and thus lose information about cell-to-cell variation. To fully understand the molecular mechanism underlying quorum sensing as well as the general principles underlying bacterial communication and cooperation, we must study this process at the single-cell level.

**Figure 1:**
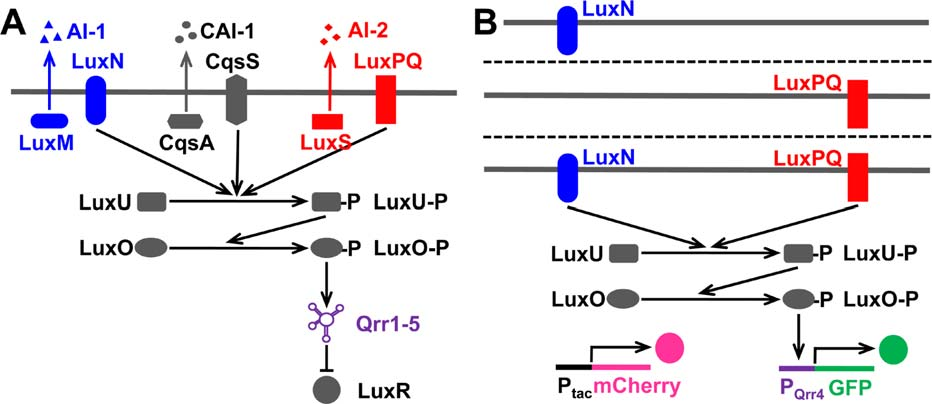
The Quorum-Sensing Circuit of Wild-Type V. harveyi and Sensor Mutants Used in These Studies (A) The wild-type quorum-sensing circuit consists of three parallel signaling pathways with three different autoinducers: AI-1, CAI-1, and AI-2. Their synthases are LuxM, CqsA, LuxS, and their transmembrane receptors are LuxN, CqsS, LuxPQ, respectively. In the absence of autoinducers (i.e., at low cell density), the receptors act predominantly as kinases and pass phosphate to LuxU and thence to LuxO. Phosphorylated-LuxO (LuxO-P) activates transcription of genes encoding five small regulatory RNAs (sRNAs). These sRNAs inhibit the translation of LuxR. In the presence of autoinducers (i.e., at high cell density), the receptors switch to a predominantly phosphatase-active state that reverses the direction of phosphoryl transfer through the circuit, so that LuxO is dephosphorylated and becomes inactive. Therefore, the genes encoding the five sRNAs are not transcribed, *luxR* mRNA is translated, and LuxR protein is made. (B) In the LuxN^+^ sensor mutant (top), the genes encoding *cqsS*, *luxPQ*, and the gene encoding the AI-1 synthase *luxM* are deleted. As a result, this mutant only responds to exogenously added AI-1. The LuxPQ^+^ sensor mutant (middle) responds exclusively to exogenous AI-2, and the LuxN^+^ LuxPQ^+^ sensor mutant (bottom) responds to exogenous AI-1 and AI-2. We quantify the responses using a *qrr4*-*gfp* transcriptional reporter fusion that is activated by LuxO-P. As an internal standard for fluorescence, the gene encoding mCherry is fused to a constitutive *tac* promoter and integrated at an intergenic region of the chromosome.

To begin to explore the above questions, we investigated the network of the model quorum-sensing bacterium Vibrio harveyi, the first bacterium shown to use more than one autoinducer for quorum sensing [15,16]. V. harveyi has a particularly ideal system in which to undertake these studies because the components of the quorum-sensing circuit have been defined (Figure 1A) and the autoinducers are known and in hand. V. harveyi produces and detects three autoinducers: AI-1 (3-hydroxybutanoyl homoserine lactone), CAI-1 ([S]-3-hydroxytridecan-4–1), and AI-2 ([2S,4S]-2-methyl-2,3,3,4-tetra hydroxytetrahydrofuran borate) [6,17,18]. AI-1 is only produced by V. harveyi, CAI-1 is produced by V. harveyi as well as other Vibrios, and as discussed, AI-2 is produced by many bacterial species. Thus AI-1, CAI-1, and AI-2 could provide information about the numbers of V. harveyi, Vibrios, and total bacteria in the vicinity, respectively. The three autoinducers are detected extracellularly by their cognate transmembrane receptors: LuxN, CqsS, and LuxPQ, respectively [19]. Information from the autoinducer-sensing pathways is transduced through shared components LuxU and LuxO [20–22] and five small regulatory RNAs (sRNAs) [23,24] to the master quorum-sensing regulator LuxR [25] (Figure 1A). LuxR activates and represses genes including those required for bioluminescence, siderophore production, type III secretion, and metalloprotease production [2,26–28].

Here we report the quantitative single-cell fluorescence-microscopy studies of V. harveyi quorum sensing, which have allowed us to define the mechanism of quorum-sensing autoinducer signal integration. Our studies revealed highly uniform behavior in individual cells, suggesting that the V. harveyi quorum-sensing circuit is designed to tightly synchronize the population response to autoinducers. This network operates in stark contrast to other regulatory circuits (e.g., such as that underpinning sporulation in Bacillus subtilis), which appear designed to generate diversity among the members of the population [29–32]. We also discovered that information from the different autoinducers is integrated in a strictly additive way, with an unexpected balance between the signaling strengths of the different autoinducers, allowing the population as a whole to distinguish multiple states of autoinducer concentration. These results have important implications for the developmental cycle of V. harveyi and possibly for other bacteria that use multiple autoinducers.

## Results

To investigate the mechanism underlying how V. harveyi integrates the information contained in its multiple autoinducers, we engineered strains that allowed us to examine each quorum-sensing signaling pathway in isolation as well as strains that allowed us to analyze the signaling properties of the combined pathways. In the present study, we focused only on integration of signals from autoinducers AI-1 and AI-2 through the LuxN and LuxPQ pathways, respectively. We did not study CAI-1 signaling through CqsA. Our rationale is as follows: First, under our laboratory conditions, the CAI-1 pathway is the weakest of the three signaling pathways, and thus AI-1 and AI-2 are the major inputs influencing quorum-sensing–controlled gene expression; second, we wanted to analyze the simplest possible case of integration of two signals. For this set of experiments, we constructed V. harveyi strains carrying only the LuxN pathway, only the LuxPQ pathway, or both pathways. In each case, the V. harveyi strains lacked the CqsS pathway. To enable quantitative measurements of signaling through the individual and combined pathways, all the strains were engineered to contain a transcriptional fusion of *gfp* fused to a quorum-sensing responsive promoter. Additionally, all of our strains constitutively produced red fluorescent protein (i.e., mCherry) that we used as an internal standard (Figure 1B) [33].

The strains used are as follows: The LuxN^+^ strain carries wild-type *luxN* on the chromosome and is deleted for *cqsS* and *luxPQ*. The strain is also deleted for *luxM*, encoding the AI-1 synthase LuxM, and is therefore exclusively responsive to exogenously added AI-1. Similarly, the LuxPQ^+^ strain is deleted for *luxN* and *cqsS* as well as *luxS* encoding the AI-2 synthase. This strain is only responsive to exogenous AI-2. The combined LuxN^+^ LuxPQ^+^ strain lacks *cqsS*, *luxM*, and *luxS*, and is responsive to exogenously supplied AI-1 and AI-2. In each strain, *gfp* is fused to the *qrr*4 promoter, which is one of the genes encoding the quorum-sensing sRNAs that are activated by LuxO-P (Figure 1B). mCherry is driven by the constitutive *tac* promoter inserted at an intergenic region of the chromosome. Because mCherry is expressed constitutively, it reports on the cell's overall protein level, including variations due to cell size and phase of the cell cycle. Normalizing the reporter green fluorescent protein (GFP) intensity by the internal standard mCherry intensity therefore provides an accurate measure of quorum-sensing receptor activity, and eliminates errors caused by uneven illumination or inaccurate segmentation of cells during image processing. The engineered V. harveyi strains were grown to steady state (Figure S1) in broth medium containing particular autoinducer concentrations. Cells were transferred to glass slides on a microscope, and phase-contrast and fluorescence snapshots were taken. Microscopy images were processed automatically by a custom computer program to obtain fluorescence intensities of individual cells. (For details, see Materials and Methods.)

### Responses of Individual Autoinducer-Detection Pathways

**Figure 2:**
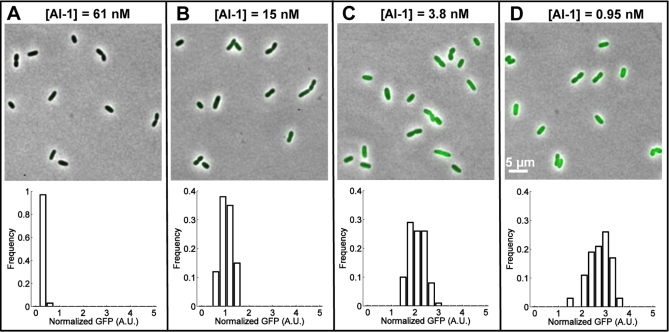
Single-Cell Microscopy Images and GFP Fluorescence Distributions of LuxN^+^ Cells of V. harveyi Snapshots of cells growing exponentially at different AI-1 concentrations (indicated above images) and the corresponding GFP fluorescence distributions of single cells. The mean GFP fluorescence intensity of each cell is normalized by the cell's mean mCherry fluorescence intensity. Each distribution is obtained from 100 cells. A.U. denotes arbitrary units.

Each autoinducer-detection pathway contributes uniquely to the overall V. harveyi integrated quorum-sensing response. Thus, to understand how cells communicate, understanding the signaling properties of the individual quorum-sensing pathway is imperative. Toward this end, we measured dose responses of individual cells of the LuxN^+^ mutant responding to AI-1. LuxN^+^ mutant cells were grown in series-diluted concentrations of exogenous AI-1, and the distributions of P_Qrr4_-GFP intensities of individual cells at each AI-1 concentration were obtained (Figure 2). A gradual increase in the mean P_Qrr4_-GFP intensity distribution occurred with decreasing AI-1 concentration, reflecting increasing kinase activity of LuxN, and, consequently, increasing LuxO-P concentration. While we observed heterogeneity in P_Qrr4_-GFP expression over the population, the distribution of P_Qrr4_-GFP intensities remained single-peaked with moderate variance around the population average at all AI-1 concentrations (cell-to-cell variation was somewhat smaller after normalizing by mCherry intensity; see Figure S2). This result suggests that all the V. harveyi cells respond identically to AI-1, which promotes well-coordinated cellular behavior across the population. The shift in the mean P_Qrr4_-GFP intensity between zero and saturating AI-1 is obviously larger than the standard deviation within the population at any AI-1 concentration, suggesting that cell-to-cell variation, or noise, in quorum sensing is low enough to allow the cells to reliably mount distinct responses to low and high AI-1 concentrations.

**Figure 3:**
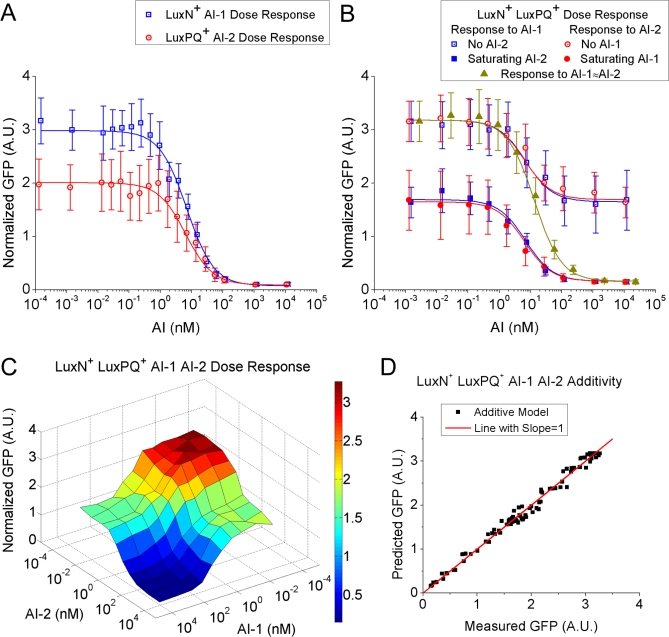
Autoinducer Dose Responses of V. harveyi Sensor Mutants (A) Dose responses of LuxN^+^ cells to AI-1 (blue) and LuxPQ^+^ cells to AI-2 (red). Each average and standard deviation (error bar) of normalized GFP was obtained from microscopy images of 100 cells. Curves were fitted using α_AI_ + β_AI_(1 + [AI]/*K*_AI_) with α_AI-1_ = 0.07, β_AI-1_ = 2.9, *K*_AI-1_ = 6.9 nM and α_AI-2_ = 0.09, β_AI-2_ = 1.9, *K*_AI-2_ = 6.4 nM. A.U. denotes arbitrary units. (B) Dose responses of LuxN^+^ LuxPQ^+^ cells to either AI-1 (blue) or AI-2 (red) while the other autoinducer is either absent (open squares and circles) or present at a saturating concentration (solid squares and circles). Data in yellow-green represent the response to approximately equal amounts of AI-1 and AI-2 (*x*-axis values indicate total autoinducer concentrations). (C) Dose–response surface of LuxN^+^ LuxPQ^+^ cells to various combinations of AI-1 and AI-2. Each vertex of the grid is the averaged normalized GFP fluorescence intensity obtained from a population of 100 cells exposed to the specified AI-1 and AI-2 concentrations. The dose–response curves in (B) correspond to cuts through this surface. (D) The response of LuxN^+^ LuxPQ^+^ cells to combined AI-1 and AI-2 shown in (C) can be well described by a simple additive model γ_0_ + γ_AI−1_/(1 + [AI-1]/*K*_AI-1_) + γ_AI-2_/(1 + [AI-2]/*K*_AI-2_), with γ_0_ = 0.16, γ_AI-1_ = 1.53, γ_AI-2_ = 1.49, *K*_AI-1_ = 6.9 nM, *K*_AI-2_ = 6.4 nM. The red line has a slope equal to one.

We performed similar individual-cell dose–response experiments on the V. harveyi LuxPQ^+^ mutant strain to determine the signaling properties of the AI-2 pathway. For comparison, in Figure 3A we show dose–response curves for both the LuxN^+^ and LuxPQ^+^ mutant strains to AI-1 and AI-2, respectively. Means and standard deviations over a population of cells are reported for each strain. Similar to the results shown in Figure 2, at all autoinducer concentrations the normalized P_Qrr4_-GFP-intensity distributions are single-peaked, with standard deviation over the mean always smaller than 0.4. For each data point, the population sample consists of 100 individual cells, thus the standard error of the mean is one-tenth of the standard deviation of the population. Each dose–response curve can be described by a simple Hill function α_AI_ + β_AI_/(1 + [AI]/*K*_AI_) with Hill coefficient equal to one. The inhibition constants for AI-1 and AI-2 are *K*_AI-1_ = (6.9 ± 0.5) nM and *K*_AI-2_ = (6.4 ± 0.5) nM, respectively. Note that a 1 nM concentration is approximately one molecule of autoinducer in the volume of a single V. harveyi cell, indicating an extremely sensitive response of V. harveyi cells to autoinducers. The LuxN^+^ strain has approximately 50% higher P_Qrr4_-GFP levels than the LuxPQ^+^ strain at low autoinducer concentrations where LuxO-P and P_Qrr4_-GFP are maximal. However, the two strains have similar residual levels of P_Qrr4_-GFP, which remain measurable above background at saturating autoinducer concentrations.

### Response of Combined Autoinducer-Sensing Pathways

The above experiments allowed us to determine the signaling response of the LuxN pathway to AI-1 and that of the LuxPQ pathway to AI-2 when each pathway is present alone. We likewise wondered how the cells respond to AI-1 and AI-2 when the two pathways are present together. To examine this, we performed experiments analogous to those above with the V. harveyi LuxN^+^ LuxPQ^+^ strain in the presence of combinations of AI-1 and AI-2. Surprisingly, we found that although the amplitudes of the autoinducer responses are different when the two quorum-sensing pathways are present individually (Figure 3A), the amplitudes of the AI-1 and AI-2 responses are nearly identical when the two pathways are present simultaneously (Figure 3B). In particular, the dose–response curves for AI-1 (blue) and AI-2 (red) almost overlap, both in the case when one autoinducer is present alone and in the case when a saturating amount of the other autoinducer is also present. Critically, the overlap of these curves depends on the extremely similar amplitudes of the responses as well as the similar inhibition constants for AI-1 and AI-2 as observed in Figure 3A. The very similar amplitudes of the two autoinducer dose–response curves demonstrate that each autoinducer-sensing pathway contributes approximately half of the total response.

Figure 3B clearly shows that when both pathways are present (e.g., in the LuxN^+^ LuxPQ^+^ strain), each autoinducer alone is only capable of partial inhibition of P_Qrr4_-GFP expression. When AI-1 and AI-2 concentrations are increased together, with similar concentrations of each autoinducer present, the resulting dose–response curve of P_Qrr4_-GFP expression covers the entire dynamic range (yellow-green curve). The P_Qrr4_-GFP distribution is always single-peaked, and noise in GFP expression is always moderate, with the standard deviation over the mean no more than 40%. Again, we take this to mean that despite the existence of noise in the quorum-sensing pathway, individual cells are able to discriminate several distinct states. For example, the P_Qrr4_-GFP distributions do not substantively overlap for these three cases: when both AI-1 and AI-2 are below 1 nM, when both are around 10 nM, and when both are above 100 nM. Thus, it appears that individual V. harveyi cells can accurately determine the level of external autoinducers. This result suggests that, in principle, V. harveyi cells can not only detect low and high cell-density states with low and high autoinducer concentrations, but also some intermediate cell-density states represented by intermediate autoinducer concentrations.

To obtain a more comprehensive view of the autoinducer response of the LuxN^+^ LuxPQ^+^ strain, we explored a grid of possible combinations of AI-1 and AI-2 concentrations. In this way, the complete dose–response surface was obtained (Figure 3C). This surface, displaying average P_Qrr4_-GFP production, is almost mirror-symmetric with respect to the equal AI-1 and AI-2 diagonal; i.e., the P_Qrr4_-GFP expression is almost invariant with respect to exchange of AI-1 and AI-2 concentrations. Notably, there are at least three distinct states of the output P_Qrr4_-GFP level: high (both AI-1 and AI-2 concentrations are low, indicated by the red area in Figure 3C), intermediate (one autoinducer concentration is low and the other is high, indicated by the two green areas), and low (both AI-1 and AI-2 concentrations are high, indicated by the blue area). This surface confirms that more than two quorum-sensing states can be deciphered by the cells. However, interestingly, under these conditions, high AI-1/low AI-2 is apparently not distinguished from low AI-1/high AI-2 (see Discussion).

### The Two Autoinducer Inputs Are Integrated Additively

For a signal-integration circuit such as the quorum-sensing circuit in V. harveyi that involves multistep, bidirectional, biochemical reactions, one might expect the two signals to be integrated in a complicated nonlinear manner. Surprisingly, however, we found quite the opposite. That is, AI-1 and AI-2 signal integration is simply additive. The dose–response surface of the LuxN^+^ LuxPQ^+^ strain can be accurately described by the additive function


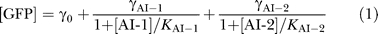


where the γ's and *K*'s are fitting parameters. The inhibition constants have the same values as in the individual pathways: *K*_AI-1_ = 6.9 nM and *K*_AI-2_ = 6.4 nM (Figure 3A and 3B). As shown in Figure 3D, the average P_Qrr4_-GFP expression values obtained from Equation 1 agree with the measured values over the entire dose–response surface. The two noncooperative Hill functions correspond to the individual responses of the LuxN and the LuxPQ pathways, respectively. Therefore, we conclude that LuxN and LuxPQ make independent, additive contributions to GFP levels presumably via additive contributions to LuxO-P.

### The Two Autoinducer Pathways Contribute Differently to Noise

**Figure 4:**
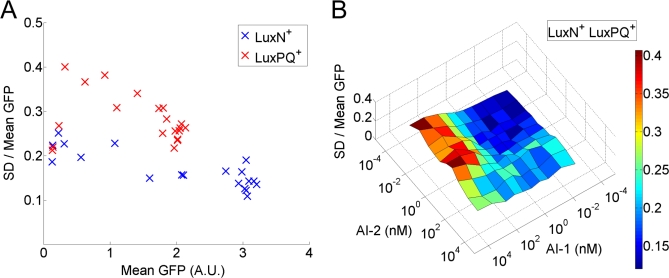
The Two Autoinducer-Sensing Pathways Contribute Differently to GFP-Expression Noise (A) Relative noise, i.e., the standard deviation (SD) of the population divided by the mean, versus mean-normalized GFP fluorescence intensity for LuxN^+^ cells at different AI-1 concentrations (blue) and for LuxPQ^+^ cells at different AI-2 concentrations (red). (B) Relative noise for LuxN^+^ LuxPQ^+^ cells as a function of AI-1 and AI-2 concentrations.

Although the two autoinducer signals are combined additively with approximately equal weights in their input to the circuit, we find that the two pathways contribute differently to the noise in P_Qrr4_-GFP expression. As shown in Figure 4A, the LuxPQ^+^ strain (with no LuxN receptor) has significantly larger relative noise, i.e., larger cell-to-cell variation, than does the LuxN^+^ strain (with no LuxPQ receptor) for the same mean P_Qrr4_-GFP level. Apparently, signaling through the LuxPQ receptor introduces more noise to the circuit than does signaling through the LuxN receptor. This difference is confirmed by the distinct noise levels observed for the LuxN^+^ LuxPQ^+^ strain treated with either saturating AI-1 or saturating AI-2 (Figure 4B). In the LuxN^+^ LuxPQ^+^ strain, the mean P_Qrr4_-GFP levels are nearly identical under these two conditions, but the relative noise is almost a factor of two larger when only LuxPQ contributes kinase activity (AI-1 saturating) than when only LuxN contributes kinase activity (AI-2 saturating). Indeed, as shown in Figure 4B, noise in the LuxN^+^ LuxPQ^+^ strain is at its absolute maximum when only LuxPQ contributes kinase activity.

### The Kinase Activities of LuxN and LuxPQ Are Regulated by Autoinducers

Our observation that the LuxN and LuxPQ pathways contribute independently and additively to P_Qrr4_-GFP expression implies that the kinase activities of LuxN and LuxPQ must be regulated by the autoinducers. We draw this conclusion from the following simple model for the signaling pathway leading to P_Qrr4_-GFP expression: We assume that LuxN and LuxPQ are the dominant kinases and phosphatases for LuxU, that phosphotransfer between LuxU and LuxO is reversible, and that P_Qrr4_-GFP expression is a linear function of LuxO-P concentration [O-P]. The final assumption follows from the observed additivity of P_Qrr4_-GFP expression with respect to AI-1 and AI-2, which is difficult to understand unless [O-P] is in the linear regime of the *qrr*4 promoter driving *gfp*, i.e., the maximal [O-P] is far below the level required to half saturate the promoter activity. The kinetic equations describing this model are


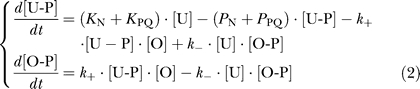


where [U-P] is the LuxU-P concentration, and *K*_N_, *K*_PQ_, *P*_N_, and *P*_PQ_ are the total cellular kinase and phosphatase activities of LuxN and LuxPQ, respectively. At steady state, the time derivatives in Equation 2 can be set to zero, yielding


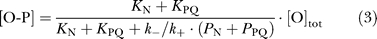


where [O]_tot_ is the total concentration of LuxO. To explain the observed broad range of additivity of P_Qrr4_-GFP expression with respect to the autoinducers, Equation 3 must be separable into two terms, one of which depends only on AI-1 and the other only on AI-2. This is possible if the autoinducers regulate the receptor kinase activities *K*_N_ and *K*_PQ_, but not if the autoinducers regulate only the receptor phosphatase activities *P*_N_ and *P*_PQ_, since the latter appear only in the denominator of Equation 3. Indeed, for additivity to be achieved, the denominator of Equation 3 must be approximately constant, which implies one of two scenarios: (1) only the kinase activities of LuxN and LuxPQ are regulated by autoinducers while phosphatase activities are not, and the kinase and phosphatase activities satisfy *K*_N_ + *K*_PQ_ << *k*_−_/*k*_+_ · (*P*_N_ + *P*_PQ_), implying that LuxO-P levels are far from saturation, i.e., [O‐P] << [O]_tot_; and (2) the kinase and phosphatase activities are both regulated, but their sum is independent of autoinducer concentration such that *K*_N_ + *K*_PQ_ + *k*_−_/*k*_+_ · (*P*_N_ + *P*_PQ_) remains constant. Unlike the first scenario, the second scenario requires fine-tuning of reaction rates and therefore seems less likely. While the signaling pathways leading to LuxO-P are likely to include some processes not considered in our simple model (e.g., intrinsic dephosphorylation of LuxU-P and LuxO-P), our qualitative conclusions—in particular that the kinase activities of LuxN and LuxPQ must be autoinducer regulated—are robust to such quantitative corrections.

Since the amplitudes of the responses to AI-1 and AI-2 are almost identical in the LuxN^+^ LuxPQ^+^ strain (Figure 3B), the maximum total kinase activities of the two receptors LuxN and LuxPQ must be nearly the same (i.e., *K*_N_ 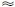 *K*_PQ_). However, for the strains expressing only a single receptor type, the peak P_Qrr4_-GFP expression is 50% higher for the LuxN^+^ than for the LuxPQ^+^ strain (Figure 3A). This apparent discrepancy can be readily accounted for if the total phosphatase activity of LuxPQ is higher than that of LuxN, i.e., *P*_PQ_ > *P*_N_ (including possible differences in receptor concentration).

## Discussion

Living cells monitor their environment using a variety of signal-transduction systems, ranging from simple two-component systems in prokaryotes to highly complex signal-transduction networks in mammalian cells. Since environmental cues are always numerous, the ability to integrate multiple signals is indispensable if cells are to behave appropriately. However, the mechanisms and logic by which cells integrate environmental signals remain, by and large, poorly understood. Here we have quantitatively analyzed the integration of multiple autoinducer signals by the model quorum-sensing bacterium V. harveyi using single-cell fluorescence microscopy. Our studies reveal a unified response across the population, with moderate cell-to-cell variation. We find that signals from two distinct autoinducers, AI-1 and AI-2, are combined strictly additively in a single phosphorelay pathway, with each autoinducer contributing nearly equally to the total response. Moreover, the cell-to-cell variation in response is small enough that the entire population of cells can reliably distinguish at least three distinct conditions of external autoinducer concentration.

We used GFP under the control of the chromosomal sRNA Qrr4 promoter as a reporter of the activity of the quorum-sensing signaling pathway (Figure 1). In all our strains, the GFP distribution was always single-peaked at all autoinducer concentrations, with cell-to-cell standard deviation no more than 40% of the mean, suggesting that populations of V. harveyi cells respond coherently to autoinducer signals. By contrast, genes in some other bacterial systems are known to have bimodal (i.e., two-peaked) expression distributions. In many cases, bimodal gene expression is also hysteretic (i.e., cells remain for a long time in one state of expression), which constitutes a form of cellular “memory.” For example, bimodal distributions in gene expression enable sporulation and competence in B. subtilis [29–32], stringent response in mycobacteria [34], and induction of the *lac* operon in Escherichia coli [35,36]. In all these cases, bimodality and hysteresis are believed to provide advantages to the organism by enabling phenotypic diversity within isogenic populations. In general, hysteresis in gene expression requires some form of positive feedback. The lack of bimodality in our engineered strains of V. harveyi is expected since there is no positive-feedback loop in the circuit controlling Qrr sRNA expression in these cells. Since our engineered strains lack both the downstream transcription factor LuxR and the autoinducer synthases, there exists the possibility that the sRNAs or LuxR could feed back positively to the synthases and produce a bistable circuit in wild-type cells. In quorum sensing, bistability has only been reported for a rewired LuxIR circuit in V. fischeri [37]. In this case, the positive feedback and the resulting bistability and hysteresis occur at the population level and divide the entire population into two separate subpopulations, each with a unique phenotype. Our consistent observation of a narrowly peaked distribution of quorum-sensing responses strongly suggests that V. harveyi cells respond in unison to the presence of autoinducer signals. For quorum-sensing cells, in contrast to bacteria undergoing competence, sporulation, or the stringent response, operating as a coherent population appears to be more important than maintaining phenotypic diversity.

An outstanding question is why V. harveyi and related species use multiple autoinducer signals, but funnel all the information into a single pathway. We can envision two main possibilities (potentially in combination): The multiple autoinducers could reveal information about the community composition (e.g., which species are present and in what abundance), or the multiple autoinducers could reveal information about the development stage of the community (e.g., the growth stage of a biofilm). In support of the first possibility, the three autoinducers used by V. harveyi have distinct ranges of species specificity: intraspecies for AI-1, within Vibrios for CAI-1, and across many species for AI-2 [7]. Thus, different combinations of the three autoinducers could indicate different compositions of a bacterial community. In our experimental conditions, however, we found that cells could not distinguish between high AI-1/low AI-2 and high AI-2/low AI-1 (Figure 3B and 3C). This result argues for the second possibility, namely that different combinations of autoinducers represent different stages of community development. For example, if a growing V. harveyi community typically accumulates AI-2 before AI-1, then the signaling contour in Figure 3C would always be traversed along the right edge, and cells could reliably interpret an intermediate signaling strength as a condition of high AI-2/low AI-1, since the opposite condition of high AI-1/low AI-2 would rarely, if ever, be encountered. In much of eukaryotic development (e.g., embryogenesis), the rate of development is fixed and driven by a clock [38], obviating the need for a signal representing the stage of development. However, without the support of a surrounding organism, the rate of development of a bacterial community depends on unpredictable environmental conditions, such as nutrient availability, and therefore some means of determining the stage of development is required so that cells in the community can behave appropriately. Recent models of biofilm growth suggest that communities may be mixed at early stages, but that at later stages competition for nutrients by overgrowth of neighboring cells can result in large domains of cells descended from a single progenitor, and therefore composed of a single species [39]. If so, generic signals such as AI-2 may be most informative at early stages of biofilm growth, while species-specific signals such as AI-1 may be reserved for later stages. We are currently exploring the order of accumulation of the V. harveyi autoinducers AI-1, CAI-1, and AI-2 to test whether different autoinducer combinations could signal different stages of community development.

Given that the autoinducer signals are combined in one pathway in V. harveyi, why should the signals be combined additively, as we observe for AI-1 and AI-2? Simple alternatives would be for saturating autoinducer levels to be combined in “logic gates,” such as AND, in which both autoinducer signals would be required for a full response, or OR, in which either signal would be sufficient for a full response. However, these logic gates have only two possible output states: on or off. In contrast, the addition of the two autoinducer signals allows for more than two output states of the signaling pathway, and therefore potentially allows for more than two expression states of quorum-sensing regulated genes. Indeed, we discovered three distinct levels of signaling strength, represented by the heights of the plateaus in Figure 3C. Moreover, the standard deviation of P_Qrr4_-GFP expression across the population of cells was sufficiently small (Figure 4B) so that the entire population can apparently distinguish the three distinct plateau heights. This means that, in principle, every cell in the population can distinguish three external autoinducer conditions: both autoinducers low, both autoinducers high, and a third condition in which one autoinducer is high and the other is low. The reliability with which cells can distinguish among these three conditions is increased by the equal spacing of the plateau heights as shown in Figure 3C. Given a uniformly distributed input of autoinducer concentration and the observed level of noise (i.e., cell-to-cell variation in P_Qrr4_-GFP expression), a significantly unequal spacing of the plateau heights would lead to overlapping distributions of P_Qrr4_-GFP expression for the two more closely spaced plateaus. The implication is that noise might then cause some cells to misinterpret external conditions and regulate quorum-sensing genes inappropriately. The need for all cells to reliably distinguish among multiple autoinducer conditions may therefore explain not only the additivity of the quorum-sensing pathway, but also why the contributions of the AI-1 sensor LuxN and the AI-2 sensor LuxPQ to the total kinase activity are so nearly equal—equal kinase activities mean equally spaced plateau heights, which in turn mean that individual cells are less likely to confuse one autoinducer condition with another.

The existence of multiple quorum-sensing output states potentially underpins diverse patterns of quorum-sensing regulated gene expression. For example, in previous studies, the quorum-sensing circuit of V. harveyi was found to act as an autoinducer “coincidence detector” (i.e., requiring both AI-1 and AI-2) for full induction of bioluminescence [19,40]. Thus, in the present context, the three distinguishable levels of signaling output (indicated by Qrr4 promoter activity) appear to be collapsed by downstream signal-processing events to two levels of bioluminescence. More generally, the target genes of quorum sensing could be tuned to different signaling output levels so that only particular classes of genes are switched ON/OFF at early, middle, or late stages of community development. Alternatively, some genes could have graded expression between these different developmental stages. The requirement for multiple distinct output states might also explain our observation of a graded, rather than switch-like, response of the Qrr4 promoter. Specifically, our dose–response data are well described by a noncooperative, *n* = 1 Hill function response to both autoinducers. Cooperativity would have resulted in an *n* > 1 Hill function and therefore a more switch-like response of P_Qrr4_-GFP to autoinducers. During the signaling process, cooperativity could in principle have arisen from the binding of autoinducers to receptors, transfer of phosphate among the protein components in the phosphorelay, and/or binding of phosphorylated LuxO to DNA. Our results suggest that in fact all of these steps are noncooperative, despite the fact that the receptors are likely dimers [22] and that LuxO may function as a tetramer or octamer [Tu KC, unpublished data]. Indeed, a graded noncooperative response of Qrr expression to autoinducers is essential for the existence of multiple, distinguishable quorum-sensing states, as a switch-like response of the Qrr expression would have allowed for only two states.

Based on a simple kinetic model for signaling (Equation 2), we have argued that the kinase activities of LuxN and LuxPQ are regulated by autoinducers, whereas for most two-component receptors, it is still an open question whether the kinase or phosphatase or both activities are regulated by input stimuli. Previously, LuxN receptors have been successfully modeled as switching between two states: the ON (kinase dominant) and OFF (phosphatase dominant) states [41,42]. Each receptor has intrinsic kinase and phosphatase rates depending only on the state in which the receptor exists. Extending this model to LuxPQ, the total cellular kinase activities *K*_N_ and *K*_PQ_ consist of a major contribution from those receptors in the ON state with little or no contribution from those in the OFF state. From the constraints set by additivity, we conclude that the phosphatase activities *P*_N_ and *P*_PQ_ are unregulated (i.e., receptors have the same phosphatase rates in both the ON and OFF states). Note that autoinducer concentrations only affect the thermal balance between ON and OFF states, and therefore the kinase and phosphatase activities are regulated only via the biasing of receptors between states (of course, the total kinase and phosphatase activities also depend on receptor concentrations). The low levels of P_Qrr4_-GFP expression with saturating AI-1 in the LuxN^+^ strain, saturating AI-2 in the LuxPQ^+^ strain, and saturating AI-1 plus AI-2 in the LuxN^+^ LuxPQ^+^ strain indicate that kinase rates in the OFF states are much smaller than those in the ON states for both LuxN and LuxPQ. By decreasing the fraction of receptors in the ON state, autoinducers reduce the total kinase activity of the quorum-sensing receptors in V. harveyi. (See Text S1 for more details.)

Regulation of the kinase activities of LuxN and LuxPQ appears to be necessary to achieve three equally spaced levels of LuxO-P (Equation 3). The requirement for kinase regulation in V. harveyi quorum sensing therefore appears to stem from the need to combine multiple input signals into more than two distinguishable output levels of LuxO-P. One prediction from this analysis is that the sensor CqsS, which was not present in our strains, is likely to also have its kinase activity regulated by its autoinducer CAI-1. Moreover, CqsS is likely to contribute additively to total kinase activity and with a strength comparable to that of LuxN and LuxPQ, resulting in four maximally distinguishable levels of kinase activity and therefore four distinguishable autoinducer conditions.

The similarity of the responses to AI-1 and AI-2 is striking, not only in the amplitudes but also in the inhibition constants. We speculate that V. harveyi usually encounters similar amounts of AI-1 and AI-2, and the responses of receptors have been optimized to match the natural dynamic range of autoinducer concentrations. It has been demonstrated that single mutations in the receptors LuxN and LuxPQ can result in dramatic changes in their inhibition constants [22,42], so the similar values for AI-1 and AI-2 may represent an evolved optimum.

We also quantified the noise in P_Qrr4_-GFP expression in our three reporter strains. Noise is an inherent feature of signal transduction and gene expression both in prokaryotes and eukaryotes. Due to the low copy number of cellular components and the stochastic nature of biochemical reactions, fluctuations are inevitable. Large fluctuations might be deleterious for processes requiring precise control but beneficial for those providing phenotypic diversity. In quorum sensing, bacterial cells detect population cell density to coordinate their behavior on a community-wide scale. Low noise in quorum-sensing signal transduction might therefore benefit the population of cells by allowing all cells to behave correctly and in unison at each stage of community development. Indeed, we observed low noise in P_Qrr4_-GFP expression in all our strains. At all autoinducer concentrations the standard deviation over the mean was less than or close to 0.4 (Figure 4). In other systems, the dominant source of cell-to-cell variation in gene expression has been attributed to extrinsic noise, e.g., differences among cells in concentrations of general purpose cellular components such as RNA polymerases and ribosomes [8]. In the quorum-sensing circuit we have studied, the noise we observed is also likely due to extrinsic factors rather than to biochemical noise in phosphotransfer or transcription and translation of P_Qrr4_-GFP. The most likely source of the noise we observed is fluctuations in concentrations of the pathway components, such as the receptors LuxN and LuxPQ and the response regulator LuxO. The noisier response in LuxPQ pathway is very likely caused by variations in the copy number of the LuxPQ receptors, which suggests that there could be some additional regulation of receptor expression in the quorum-sensing circuit.

## Materials and Methods

### Bacterial strains and media.

All V. harveyi strains used in this study were derived from the wild-type strain BB120 [43] and grown aerobically at 30 °C in Autoinducer Bioassay (AB) broth. E. coli S17–1λ*pir* was used for general DNA manipulation and grown with aeration at 37 °C in LB (Luria-Bertani) broth. The relevant strains and plasmids are listed in Table S1.

### DNA manipulations.

DNA manipulation was performed using standard procedures [44]. Phusion DNA polymerase was used for PCR reactions. dNTPs, restriction enzymes, and T4 DNA ligase were obtained from New England Biolabs. DNA purification kits were provided by Qiagen. E. coli was transformed by electroporation using a Bio-Rad Micro Pulser. Plasmids were introduced into V. harveyi by conjugation [15] and exconjugants were selected using the antibiotic resistances carried on the plasmids together with polymyxin B.

### Fluorescent protein reporter construction.

A *cat*-resistance cassette from pKD3 [45] was cloned into vector pCMW1 [7] downstream of *gfp* at the BamH1 site, making pTL3. The GFP-Cm^r^ fragment from this construct was subsequently amplified by PCR and recombined using the λ red technique [45] into a cosmid to replace the wild-type *qrr*4 gene, producing pTL20. Lastly, P_Qrr4_-GFP-Cm^r^ was introduced onto the chromosome to replace *qrr*4 by allelic recombination. Ptac-mCherry was amplified from the vector pEVS143-mCherry containing an IPTG inducible mCherry gene and cloned into pKD13 [45] at the NheI site, resulting in pTL82. The cosmid, pTL83, was constructed using the λ red technique by recombining the Ptac-mCherry-Kan^r^ fragment into the intergenic region downstream of the entire *lux* operon. Final insertion of Ptac-mCherry-Kan^r^ onto the V. harveyi chromosome was accomplished by allelic recombination.

### V. harveyi strain construction.

To construct the various V. harveyi sensor mutants, pKM780 carrying Δ*luxS*::Cm^r^, pJMH291 carrying Δ*luxN*::Cm^r^, pDLS100 carrying Δ*luxPQ*::Cm^r^, pJMH244 carrying Δ*cqsS*::Cm^r^, and pKM705 carrying Δ*luxR*::Kan^r^ were used to sequentially delete the corresponding wild-type genes by allelic recombination. Following each gene deletion, the plasmid pTL18 containing an IPTG-inducible FLP recombinase, derived from pEVS143 and pCP20 [45], was introduced into the V. harveyi strain to eliminate the antibiotic resistance marker on the chromosome.

### Fluorescence assays.

For dose–response experiments, V. harveyi strains LuxN^+^ (TL87), LuxPQ^+^ (TL88), and LuxN^+^ LuxPQ^+^ (TL89) were grown in AB medium for 8∼12 h. Growth was monitored by measuring optical density at 600 nm. Cultures were diluted to OD_600_ = 10^−6^ ∼ 10^−7^, and exogenous autoinducers were added at the specified concentrations. Following growth to steady state (13∼14 h; OD_600_ = 0.005 ∼ 0.05), cells were concentrated by centrifugation and maintained on ice until measurements were made. One μl of cell culture was spread on a glass slide and covered with a 1% AB agarose pad as well as a coverslip.

### Microscopy.

Phase-contrast and fluorescent images were taken at room temperature using a Nikon TE-2000U inverted microscope. Custom Basic code was written to control the microscope. Images were acquired using a 100× oil objective and a cooled CCD camera (−65 °C, Andor iXon). Segmentation of individual cells was performed on phase-contrast images. Background and cellular auto-fluorescence values were subtracted from the green and red channels, respectively. Total fluorescence intensity of each cell was obtained by summing all pixels and fractions of pixels in the segmented cell region. Normalized GFP values for each cell were calculated by normalizing total green to total red fluorescence intensity.

## Supporting Information

Figure S1 The Engineered V. harveyi Strains Were Grown to Steady State Sensor mutants LuxN^+^ (blue squares), LuxPQ^+^ (red circles), and LuxN^+^ LuxPQ^+^ (black triangles) were grown in AB medium at 30 °C. At time zero, cell cultures were diluted into fresh AB medium to OD_600_ = 10^−7^ ∼10^−6^. After 12-h growth, cell samples were collected for snapshots under the microscope. For each data point, 100 cells were measured, and the means (symbols) and standard deviations (error bars) of normalized GFP are plotted. Apparently, under the specified conditions, cells are in steady state in P_Qrr4_-GFP expression between 12 and 14 h.
(34 KB TIF)

Figure S2 Cell-to-Cell Variation in P_Qrr4_-GFP Expression Is Smaller after Normalizing by mCherry Intensity (A) Cell-to-cell variation, represented by relative noise, i.e., the standard deviation (SD) of the population divided by the mean, versus mean GFP intensity for LuxN^+^ (blue crosses), LuxPQ^+^ (red pluses), and LuxN^+^ LuxPQ^+^ (black dots) cells at different autoinducer concentrations.
(B) Cell-to-cell variation for the same cell samples as in (A), but with the GFP intensity of each cell normalized by the same cell's mCherry intensity. Cell-to-cell variation (relative noise) is smaller after normalization.
(254 KB TIF)

Table S1 V. harveyi Strains and Plasmids Used in This Study (64 KB DOC)

Text S1 Constraints on Kinase and Phosphatase Rates Set by Additivity (44 KB PDF)

## Abbreviations

AI: autoinducer
GFP: green fluorescent protein
sRNA: small RNA
