# Supplementary figures and images for: Quantifying the Integration of Quorum-Sensing Signals with Single-Cell Resolution
Source: PLoS Biol. 2009 Mar 24;7(3):e1000068. doi: 10.1371/journal.pbio.1000068 (PMC2661960; doi:10.1371/journal.pbio.1000068)

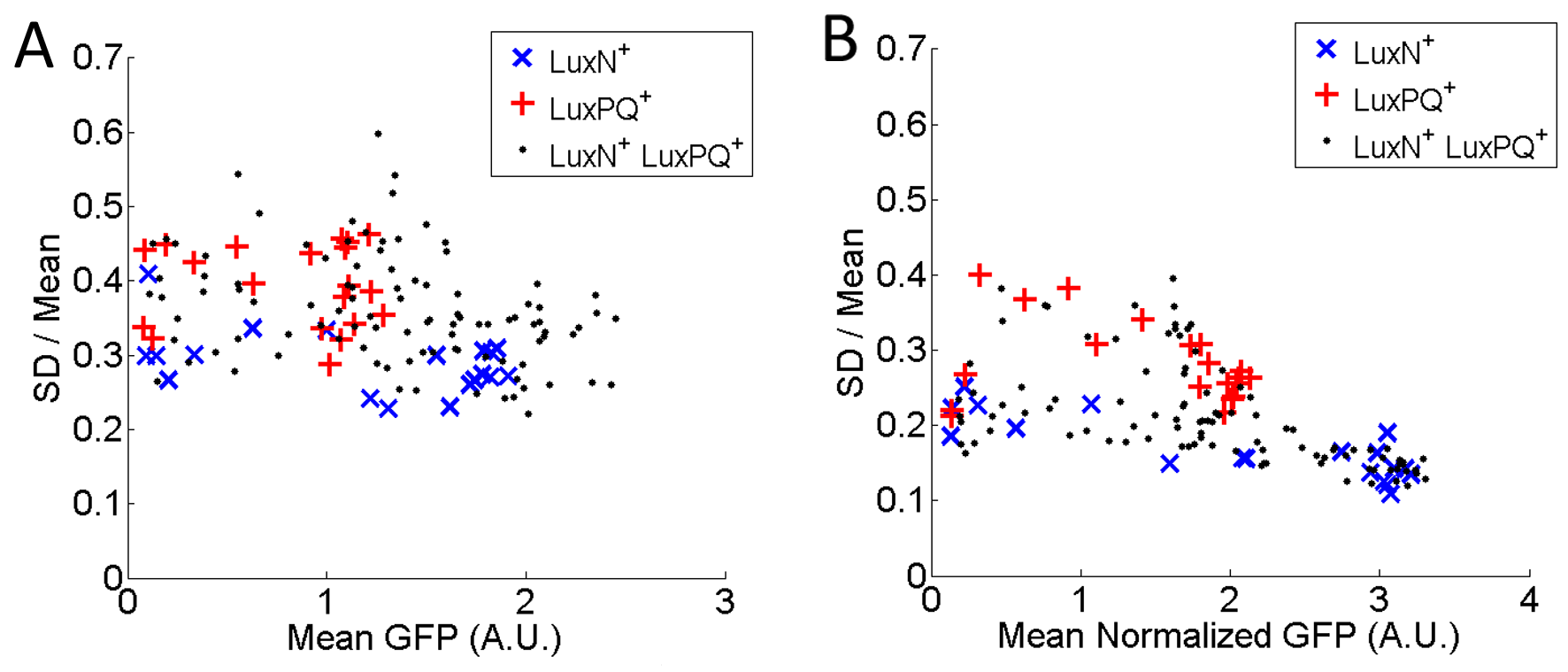

Supplement: Figure S2 — (A) Cell-to-cell variation, represented by relative noise, i.e., the standard deviation (SD) of the population divided by the mean, versus mean GFP intensity for LuxN+ (blue crosses), LuxPQ+ (red pluses), and LuxN+ LuxPQ+ (black dots) cells at different autoinducer concentrations. (B) Cell-to-cell variation for the same cell samples as in (A), but with the GFP intensity of each cell normalized by the same cell's mCherry intensity. Cell-to-cell variation (relative noise) is smaller after normalization. (254 KB TIF) [file pbio.1000068.sg002.tif]
