# Supplementary material for: Quantifying the Integration of Quorum-Sensing Signals with Single-Cell Resolution
Source: PLoS Biol. 2009 Mar 24;7(3):e1000068. doi: 10.1371/journal.pbio.1000068 (PMC2661960; doi:10.1371/journal.pbio.1000068)
Supplement: Table S1 — (64 KB DOC) [file pbio.1000068.st001.doc]

**Table S1. *V. harveyi*** Strains and Plasmids Used in this Study.

| **Strain** | **Relevant genotype** | **Reference or source** |
| --- | --- | --- |
| BB120 | Wild-type | 1 |
| JMH363 | Δ*luxM* | 2 |
| KM816 | Δ*luxS* | 2 |
| TL14 | Δ*luxM* Δ*luxPQ* | This study |
| TL15 | Δ*luxN* Δ*luxS* | This study |
| TL16 | Δ*luxM* Δ*luxS* | This study |
| TL25 | Δ*luxM* Δ*luxPQ* Δ*cqsS* | This study |
| TL26 | Δ*luxN* Δ*luxS* Δ*cqsS* | This study |
| TL27 | Δ*luxM* Δ*luxS* Δ*cqsS* | This study |
| TL28 | Δ*luxM* Δ*luxPQ* Δ*cqsS* Δ*luxR*::Kan^r^ | This study |
| TL29 | Δ*luxN* Δ*luxS* Δ*cqsS* Δ*luxR*::Kan^r^ | This study |
| TL30 | Δ*luxM* Δ*luxS* Δ*cqsS* Δ*luxR*::Kan^r^ | This study |
| TL43 | Δ*luxM* Δ*luxPQ* Δ*cqsS* Δ*luxR* Δ*qrr4*::*gfp* | This study |
| TL44 | Δ*luxN* Δ*luxS* Δ*cqsS* Δ*luxR* Δ*qrr4*::*gfp* | This study |
| TL45 | Δ*luxM* Δ*luxS* Δ*cqsS* Δ*luxR* Δ*qrr4*::*gfp* | This study |
| TL87 | Δ*luxM* Δ*luxPQ* Δ*cqsS* Δ*luxR* Δ*qrr4*::*gfp* Ptac-*mCherry*-Kan^r^ | This study |
| TL88 | Δ*luxN* Δ*luxS* Δ*cqsS* Δ*luxR* Δ*qrr4*::*gfp*  Ptac-*mCherry*-Kan^r^ | This study |
| TL89 | Δ*luxM* Δ*luxS* Δ*cqsS* Δ*luxR* Δ*qrr4*::*gfp* Ptac-*mCherry*-Kan^r^ | This study |
| **Plasmid** | **Relevant features** | **Reference or source** |
| pKM780 | pLAFR2 with Δ*luxS*::Cm^r^, Tet^r^ | Mok KC unpublished |
| pJMH291 | pLAFR2 with Δ*luxN*::Cm^r^, Tet^r^ | Henke JM unpublished |
| pDLS100 | pLAFR2 with Δ*luxPQ*::Cm^r^, Tet^r^ | 3 |
| pJMH244 | pLAFR2 with Δ*cqsS*::Cm^r^, Tet^r^ | 4 |
| pKM705 | pLAFR2 with Δ*luxR*::Kan^r^, Tet^r^ | Mok KC unpublished |
| pPH1J1 | Broad host range plasmid to kick out pLAFR, Gent^r^ | 5 |
| pQrr4 | pLAFR2 with *qrr4* locus, Tet^r^ | 6 |
| pBB1 | pLAFR2 with *luxCDABE* locus, Tet^r^ | 7 |
| pCMW1 | PCR template for GFP, Cm^r^ | 2 |
| pEVS143-mCherry | IPTG inducible mCherry, Kan^r^ | 8 & Tu KC unpublished |
| pCP20 | Ts FLP recombinase plasmid, Amp^r^, Cm^r^ | 9 |
| pKD3 | PCR template for Cm^r^ flanked by FRT sites, Amp^r^ | 9 |
| pKD13 | PCR template for Kan^r^ flanked by FRT sites, Amp^r^ | 9 |
| pTL3 | PCR template for GFP-Cm^r^, Kan^r^ | This study |
| pTL17 | IPTG inducible FLP recombinase, Cm^r^ | This study |
| pTL18 | IPTG inducible FLP recombinase, Tet^r^ | This study |
| pTL20 | pLAFR2 with Δ*qrr4*::*gfp*-Cm^r^, Tet^r^ | This study |
| pTL82 | PCR template for Ptac-mCherry-Kan^r^, Amp^r^ | This study |
| pTL83 | pLAFR2 with Ptac-mCherry-Kan^r^ in the intergenic region downstream of *luxCDABE* locus | This study |

**References**

1. Bassler BL, Greenberg EP, Stevens AM (1997) Cross-species induction of luminescence in the quorum-sensing bacterium *Vibrio harveyi*. *J Bacteriol* 179:4043-4045.
2. Waters CM, Bassler BL (2006) The *Vibrio harveyi* quorum-sensing system uses shared regulatory components to discriminate between multiple autoinducers. *Genes and Dev* 20: 2754-2767.
3. Neiditch MB, Federle MJ, Pompeani AJ, Kelly RC, Swem DL, Jeffrey PD, Bassler BL, Hughson FM (2006) Ligand-induced asymmetry in histidine sensor kinase complex regulates quorum sensing. *Cell* 126:1095-1108.
4. Henke JM, Bassler BL (2004) Three parallel quorum-sensing systems regulate gene expression in *Vibrio harveyi.* *J Bacteriol* 186:6902-6914.
5. Beringer JE, Beynon JL, Buchanan-Wollaston AV, Johnston AWB (1978) Transfer of the drug resistance transposon Tn5 to *Rhizobium*. *Nature* 276:633-634.
6. Tu KC, Bassler BL (2007) Multiple small RNAs act additively to integrate sensory information and control quorum sensing in *Vibrio harveyi*. *Genes and Dev* 21:221-233.
7. Bassler BL, Wright M, Showalter RE, Silverman MR (1993) Intercellular signaling in *Vibrio harveyi*: sequence and function of genes regulating expression of luminescence. *Mol Microbiol* 9:773-786.
8. Shaner NC, Campbell RE, Steinbach PA, Giepmans BNG, Palmer AE, Tsien RY (2004) Improved monomeric red, orange and yellow fluorescent proteins derived from *Discosoma* sp. red fluorescent protein. *Nature* 22:1567-1572.
9. Datsenko KA, Wanner BL (2000) One-step inactivation of chromosomal genes in *Escherichia coli* K-12 using PCR products. *Proc Natl Acad Sci USA* 97:6640-6645.
