# Supplementary material for: Quantifying the Integration of Quorum-Sensing Signals with Single-Cell Resolution
Source: PLoS Biol. 2009 Mar 24;7(3):e1000068. doi: 10.1371/journal.pbio.1000068 (PMC2661960; doi:10.1371/journal.pbio.1000068)
Supplement: Text S1 — (44 KB PDF) [file pbio.1000068.sd001.pdf]

Constraints on Kinase and Phosphatase Rates Set by Additivity

Our discovery that the signals from the two autoinducers AI-1 and AI-2 are integrated in a strictly additive way in the *V. harveyi* quorum-sensing circuit sets strong constraints on the parameters of the biochemical reactions within the circuit. Our analysis of these reactions is based on the two-state model for the quorum-sensing receptors [1,2]. Each of the receptors LuxN and LuxPQ are considered to have two distinct conformational states denoted as ON and OFF states. Associated with each state, there is an intrinsic kinase rate and an intrinsic phosphatase rate per receptor (the kinase rates are denoted q_{ON}^{+} and q_{OFF}^{+} , and the phosphatase rates are q_{ON}^{-} and q_{OFF}^{-} for the ON and OFF states). For each type of receptor, LuxN or LuxPQ, the fraction of receptors in the ON state (which equals the probability of any given receptor being in the ON state) is

$$p_{\mathrm{ON}} = \frac{1}{1+e^{F}}, \qquad \text{(S1)}$$

where

$$F = E_{\mathrm{ON}} - E_{\mathrm{OFF}} + \log\left(\frac{1+[\mathrm{AI}]/K_d^{\mathrm{OFF}}}{1+[\mathrm{AI}]/K_d^{\mathrm{ON}}}\right) \qquad \text{(S2)}$$

is the free energy difference between the ON and OFF states (all energies are in units of the thermal energy $k_{\mathrm{B}}T$). $E_{\mathrm{ON}}, E_{\mathrm{OFF}}$ are the free energies without autoinducer bound and $K_d^{\mathrm{ON}}, K_d^{\mathrm{OFF}}$ are the dissociation constants in the ON and OFF states. Note that for $K_d^{\mathrm{OFF}} << K_d^{\mathrm{ON}}$ and $E_{\mathrm{ON}} \le E_{\mathrm{OFF}}$, the fraction of receptors in the ON state can be expressed as a simple non-cooperative Hill function of autoinducer concentration

$$p_{\mathrm{ON}} = \frac{\sigma}{1+[\mathrm{AI}]/K_{\mathrm{AI}}}, \qquad \text{(S3)}$$

where $\sigma = 1\big/\left(1+e^{E_{\mathrm{ON}}-E_{\mathrm{OFF}}}\right)$ and $K_{\mathrm{AI}} = \left(1+e^{E_{\mathrm{OFF}}-E_{\mathrm{ON}}}\right)\cdot K_d^{\mathrm{OFF}}$. This was found to be the case for LuxN in a previous study (2). Autoinducers regulate the fraction of receptors in each state but not the intrinsic kinase or phosphatase rates of the ON and OFF states. The total cellular kinase and phosphatase activities of the receptors can therefore be expressed as:

$$\begin{cases} K_{\mathrm{N}} = p_{\mathrm{ON,N}}\cdot[\mathrm{N}]_{\mathrm{tot}}\cdot q^{+}_{\mathrm{ON,N}} + (1-p_{\mathrm{ON,N}})\cdot[\mathrm{N}]_{\mathrm{tot}}\cdot q^{+}_{\mathrm{OFF,N}} \\ P_{\mathrm{N}} = p_{\mathrm{ON,N}}\cdot[\mathrm{N}]_{\mathrm{tot}}\cdot q^{-}_{\mathrm{ON,N}} + (1-p_{\mathrm{ON,N}})\cdot[\mathrm{N}]_{\mathrm{tot}}\cdot q^{-}_{\mathrm{OFF,N}} \\ K_{\mathrm{PQ}} = p_{\mathrm{ON,PQ}}\cdot[\mathrm{PQ}]_{\mathrm{tot}}\cdot q^{+}_{\mathrm{ON,PQ}} + (1-p_{\mathrm{ON,PQ}})\cdot[\mathrm{PQ}]_{\mathrm{tot}}\cdot q^{+}_{\mathrm{OFF,PQ}} \\ P_{\mathrm{PQ}} = p_{\mathrm{ON,PQ}}\cdot[\mathrm{PQ}]_{\mathrm{tot}}\cdot q^{-}_{\mathrm{ON,PQ}} + (1-p_{\mathrm{ON,PQ}})\cdot[\mathrm{PQ}]_{\mathrm{tot}}\cdot q^{-}_{\mathrm{OFF,PQ}}. \end{cases} \qquad \text{(S4)}$$

The steady-state LuxO-P level is determined by these kinase and phosphatase activities of LuxN and LuxPQ according to Equation 3 in the main text. Taking account Equations S4, the denominator in Equation 3 can be rewritten explicitly as

$$\begin{aligned} &K_{\mathrm{N}} + K_{\mathrm{PQ}} + k_-/k_+\cdot\left(P_{\mathrm{N}}+P_{\mathrm{PQ}}\right) = \\ &[\mathrm{N}]_{\mathrm{tot}}\cdot\left[\beta_{\mathrm{N}} + \left(\alpha_{\mathrm{N}}-\beta_{\mathrm{N}}\right)\cdot p_{\mathrm{ON,N}}\right] + [\mathrm{PQ}]_{\mathrm{tot}}\cdot\left[\beta_{\mathrm{PQ}} + \left(\alpha_{\mathrm{PQ}}-\beta_{\mathrm{PQ}}\right)\cdot p_{\mathrm{ON,PQ}}\right], \end{aligned} \qquad \text{(S5)}$$

where

$$\begin{cases} \alpha_{\mathrm{N}} = q^{+}_{\mathrm{ON,N}} + k_-/k_+\cdot q^{-}_{\mathrm{ON,N}} \\ \beta_{\mathrm{N}} = q^{+}_{\mathrm{OFF,N}} + k_-/k_+\cdot q^{-}_{\mathrm{OFF,N}} \\ \alpha_{\mathrm{PQ}} = q^{+}_{\mathrm{ON,PQ}} + k_-/k_+\cdot q^{-}_{\mathrm{ON,PQ}} \\ \beta_{\mathrm{PQ}} = q^{+}_{\mathrm{OFF,PQ}} + k_-/k_+\cdot q^{-}_{\mathrm{OFF,PQ}}. \end{cases} \qquad \text{(S6)}$$

As discussed in the main text, to achieve the observed additivity of AI-1 and AI-2 signal integration, this denominator, expressed in Equation S5, must be approximately constant, independent of AI-1 and AI-2 concentrations. Therefore, the expression on the right hand side of Equation S5 must be independent of $p_{\mathrm{ON,N}}$ and $p_{\mathrm{ON,PQ}}$, which requires $\left|\alpha_{\mathrm{N}}-\beta_{\mathrm{N}}\right| << \beta_{\mathrm{N}}$ and $\left|\alpha_{\mathrm{PQ}}-\beta_{\mathrm{PQ}}\right| << \beta_{\mathrm{PQ}}$, i.e.

$$\begin{cases} \left| q^{+}_{\mathrm{ON,N}} - q^{+}_{\mathrm{OFF,N}} + k_{-}/k_{+} \cdot \left(q^{-}_{\mathrm{ON,N}} - q^{-}_{\mathrm{OFF,N}} \right) \right| << q^{+}_{\mathrm{OFF,N}} + k_{-}/k_{+} \cdot q^{-}_{\mathrm{OFF,N}} \\ \left| q^{+}_{\mathrm{ON,PQ}} - q^{+}_{\mathrm{OFF,PQ}} + k_{-}/k_{+} \cdot \left(q^{-}_{\mathrm{ON,PQ}} - q^{-}_{\mathrm{OFF,PQ}} \right) \right| << q^{+}_{\mathrm{OFF,PQ}} + k_{-}/k_{+} \cdot q^{-}_{\mathrm{OFF,PQ}}. \end{cases} \quad \text{(S7)}$$

These conditions imply either one of two scenarios: the first one is

$$\begin{cases} q^{-}_{\mathrm{ON,N}} \approx q^{-}_{\mathrm{OFF,N}}, \quad q^{+}_{\mathrm{ON,N}} << k_{-}/k_{+} \cdot q^{-}_{\mathrm{OFF,N}} \\ q^{-}_{\mathrm{ON,PQ}} \approx q^{-}_{\mathrm{OFF,PQ}}, \quad q^{+}_{\mathrm{ON,PQ}} << k_{-}/k_{+} \cdot q^{-}_{\mathrm{OFF,PQ}}, \end{cases} \quad \text{(S8)}$$

and the second is

$$\begin{cases} q^{+}_{\mathrm{ON,N}} - q^{+}_{\mathrm{OFF,N}} \approx k_{-}/k_{+} \cdot \left(q^{-}_{\mathrm{OFF,N}} - q^{-}_{\mathrm{ON,N}} \right) \\ q^{+}_{\mathrm{ON,PQ}} - q^{+}_{\mathrm{OFF,PQ}} \approx k_{-}/k_{+} \cdot \left(q^{-}_{\mathrm{OFF,PQ}} - q^{-}_{\mathrm{ON,PQ}} \right). \end{cases} \quad \text{(S9)}$$

The first scenario (S8) corresponds to the first one discussed in the main text: the phosphatase rates in the ON and OFF states q^{-}_{ON} and q^{-}_{OFF} are approximately the same, which means the phosphatase activity $P_{\mathrm{N}} + P_{\mathrm{PQ}}$ is effectively unregulated by autoinducers; on the other hand, from the observed response to autoinducers, the kinase rate in the OFF state must be much smaller than that in the ON state, i.e. $q^{+}_{\mathrm{OFF}} << q^{+}_{\mathrm{ON}}$, and the kinase activity $K_{\mathrm{N}} + K_{\mathrm{PQ}}$ must be much smaller than the effective phosphatase activity $k_{-}/k_{+} \cdot \left(P_{\mathrm{N}} + P_{\mathrm{PQ}} \right)$, resulting in a far-from-saturated LuxO-P level, i.e. $[\mathrm{O\text{-}P}] << [\mathrm{O}]_{\mathrm{tot}}$. The second scenario (S9) corresponds to the second one mentioned in the main text: both kinase and phosphatase activities are regulated by autoinducers (with different kinase rates q^{+}_{ON}, q^{+}_{OFF} and different phosphatase rates q^{-}_{ON}, q^{-}_{OFF} in the two states), but the change in kinase activity due to autoinducer regulation, proportional to $q^{+}_{\mathrm{ON}} - q^{+}_{\mathrm{OFF}}$, just equals the negative change in the effective phosphatase activity, proportional to $k_{-}/k_{+} \cdot \left(q^{-}_{\mathrm{OFF}} - q^{-}_{\mathrm{ON}} \right)$. This condition requires fine-tuning of the reaction rates and we consider it less likely than the first scenario.

References

1. Keymer JE, Endres RG, Skoge M, Meir Y, Wingreen NS (2006) Chemosensing in *Escherichia coli*: two regimes of two-state receptors. *Proc Natl Acad Sci USA* 103: 1786-1791.

2. Swem LR, Swem DL, Wingreen NS, Bassler BL (2008) Deducing receptor signaling parameters from in vivo analysis: LuxN/AI-1 quorum sensing in *Vibrio harveyi*. *Cell* 134:461-473.
